# Effect of conventional and self-ligating brackets on periodontal health. Systematic review and meta-analysis

**DOI:** 10.4317/jced.61378

**Published:** 2024-03-01

**Authors:** Franz-Tito Coronel-Zubiate, Sara-Antonieta Luján-Valencia, Joan-Manuel Meza-Málaga, Rubén Aguirre-Ipenza, Adriana Echevarria-Goche, Eduardo Luján-Urviola, Heber Arbildo-Vega

**Affiliations:** 1 Faculty of Health Sciences, National University Toribio Rodríguez de Mendoza de Amazonas. Chachapoyas, Peru; 2 Postgraduate School, Universidad Católica de Santa María (UCSM). Arequipa, Peru; 3 Faculty of Biological and Chemical Sciences and Engineering, Universidad Católica de Santa María (UCSM). Arequipa, Peru; 4 Faculty of Health Sciences, Universidad Continental. Lima, Peru; 5 National Institute of Health. Lima, Peru; 6 Faculty of Dentistry, Universidad Andina Néstor Cáceres Velásquez. Juliaca, Perú; 7 Faculty of Dentistry, Dentistry School, Universidad San Martín de Porres. Chiclayo, Perú; 8 Faculty of Human Medicine, Human Medicine School, Universidad San Martín de Porres. Chiclayo, Perú; 9 Faculty of Health Science, Stomatology School, Universidad Alas Peruanas. Lima, Perú

## Abstract

**Background:**

To compare the effect of conventional brackets and self-ligating brackets on periodontal health.

**Material and Methods:**

A search of information up to October 2022 was carried out in the following electronic databases: PubMed/MEDLINE, Cochrane Library, Scopus, Web of Science (WoS), EMBASE, SciELO and Google Scholar. We included studies that were randomised clinical trials, dealing with conventional brackets and self-ligating brackets and their effect on periodontal health, with no language restriction and no time limit. The Risk of Bias 2 (Rob 2.0) tool was used to determine the risk of bias of the included studies. The information selected from the studies was entered and analysed with RevMan 5.3, using the mean and standard deviation with a 95% confidence interval as a measure. Finally, an analysis was performed using the GRADE system to classify the quality of the evidence and grade the strength of the recommendation.

**Results:**

The preliminary search yielded a total of 399 articles, discarding those that did not meet the selection criteria, leaving only 13 articles. The effect of conventional and self-ligating brackets on periodontal health was determined using periodontal probing depth (PPD), plaque index (PI), gingival index (GI) and bleeding index (BI), showing advantages of self-ligating brackets in PI and BI, and no differences compared to self-ligating brackets in PPD and GI.

**Conclusions:**

Self-ligating brackets probably better preserve periodontal health compared to conventional brackets regarding plaque accumulation and bleeding on probing.

** Key words:**Conventional brackets, self-ligating brackets, periodontal health, orthodontic treatment, systematic review, meta-analysis.

## Introduction

Orthodontic treatment has evolved over time, and it is common practifce to use fixed appliances with brackets as the most commonly used procedure, including conventional brackets and self-ligating brackets (1). However, the use of brackets has an effect not only on changing the position of the teeth, but also on other structures such as the temporomandibular joint (2) and dental support tissues (3).

There are alternative treatments to the use of brackets that are considered to be friendlier to the periodontium, such as clear aligners (4), however, the use of these brackets is still predominant. Self-ligating brackets have been attributed a number of advantages over conventional brackets, such as reduced friction (5), faster archwire changes (6), better fit of the archwire in the slot and greater patient comfort. Other authors compared several characteristics between conventional and self-ligating brackets, including periodontal health, but found no statistically significant differences (7).

Systemic conditions such as diabetes mellitus (8), inflammatory bowel disease (9), malnutrition (10) or pregnancy (11) have been implicated in the occurrence of periodontal disease, as well as the possible negative effect this disease may have on other health conditions, such as diabetes mellitus (8) or heart disease (12). The harmful consequences for oral and general health of periodontal disease make it a cause for concern when dental procedures are established that may trigger its onset or aggravate its condition, such as orthodontic treatment (13). Given the above, it is clear to us that it is vital to determine the real effect that bracket systems have on periodontal health. Patients seeking orthodontic treatment have increased because malocclusions affect major oral functions, and increase predisposition to periodontal disease, temporomandibular joint and masticatory musculature injuries, as well as having negative psychosocial effects and affecting quality of life (14). Orthodontic treatment after periodontal stabilisation is known to have no detrimental effect on periodontal health in adult patients with periodontal orthodontic problems (15,16).

The purpose of this systematic review and meta-analysis was to study the effect of conventional and self-ligating brackets on periodontal health.

## Material and Methods

- Protocol and registration

The protocol of the present systematic review was defined a priori by all authors and was developed following the Preferred Reporting Items for Systematic Reviews and Meta-Analyses (PRISMA) guidelines. In addition, the present protocol was registered in the Prospective International Register of Systematic Reviews (PROSPERO) under the registration number CRD42022359099.

For the design and structure of this review, the research question was developed using the PICO (population, intervention, comparison and outcome) format as detailed below:

● Population: People of all ages and both sexes who have received orthodontic treatment.

● Intervention: People who have received orthodontic treatment with self-ligating brackets.

● Comparison: People who have received orthodontic treatment with conventional brackets.

● Results: Randomised clinical trials (RCTs).

- Focused question (PICO)

Do conventional and self-ligating brackets have any effect on the periodontal health of patients undergoing orthodontic treatment?

- Research and selection of studies

For the present systematic review and meta-analysis, a literature scan of seven electronic databases PubMed/MEDLINE, Cochrane Library, Scopus, Web of Science (WoS), EMBASE, SciELO and Google Scholar was performed until October 2022; combining keywords and subject headings according to the thesaurus of each database: “periodontal”, “periodont”, “gingival”, “plaque”, “biofilm”, “bleeding”, “inflammation”, “conventional brackets”, “self-ligating brackets”, “conventional braces”, “self-ligating braces” (Table 1). In addition, a hand search of the references of the included studies was performed.

**Table 1:**
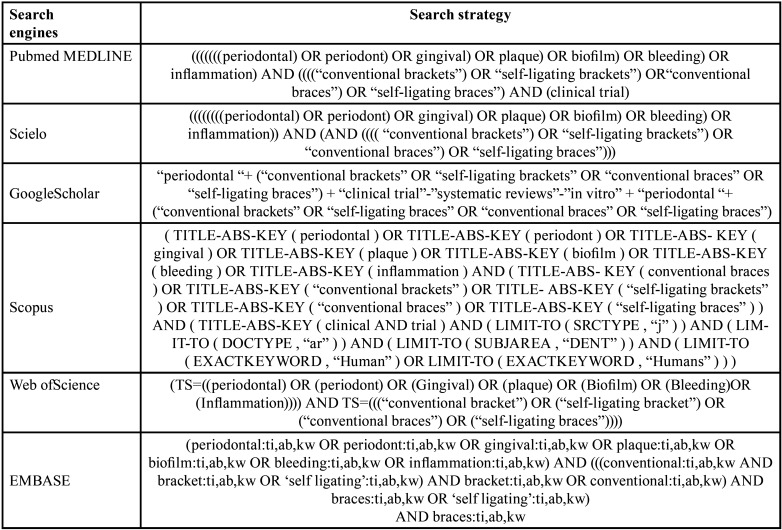
Search strategies for each search engine.

The search of the electronic database was carried out by two authors (SL, JM) independently, and included studies with the following characteristics: randomised clinical trials, studies dealing with the effect of conventional and self-ligating brackets on periodontal health, no language restriction, studies with no time limit. Systematic review, case-control and cohort articles, case reports, case series, *in vitro*, unpublished, and those reported in more than one publication with different follow-up periods were excluded.

- Data extraction

A predefined Table was used for data selection for each eligible study, including number, authors, year, study title, number of patients (male/female ratio, mean age (range), follow-up time, groups, number of patients per group, country, outcomes, inclusion criteria and exclusion criteria, periodontal probing depth (PPD), plaque index (PI), gingival index (GI) and bleeding index (BI). From each eligible study, two investigators (EL, RS) independently extracted information and all disagreements were resolved by discussion with a third reviewer (FC).

- Risk of bias assessment

The risk of bias of the included studies was assessed independently by two authors (AE, RA) calibrated (Kappa 0.85) using the Risk of Bias 2 tool (Rob 2.0). All disagreements were resolved by discussion with a third reviewer (HA). According to this tool the domains are assessed on: selection, comparability and exposure/outcomes; and then classified as: good quality, accepTable quality and low quality, according to the following parameters: randomization process, deviations from intended interventions, missing data on outcomes, outcome measurement and selection of the reported outcome.

- Analysis of results

The information selected from the studies was entered and analysed in RevMan 5.3 software (Cochrane Group, UK); using the mean and standard deviation with a 95% confidence interval as a measure. In addition, an analysis was performed using the GRADE system (GRADE Pro GDT, McMaster University and Evidence Prime Inc., Canada) for grading the quality of evidence and grading the strength of recommendation.

## Results

- Selection of studies

**Figure 1:**
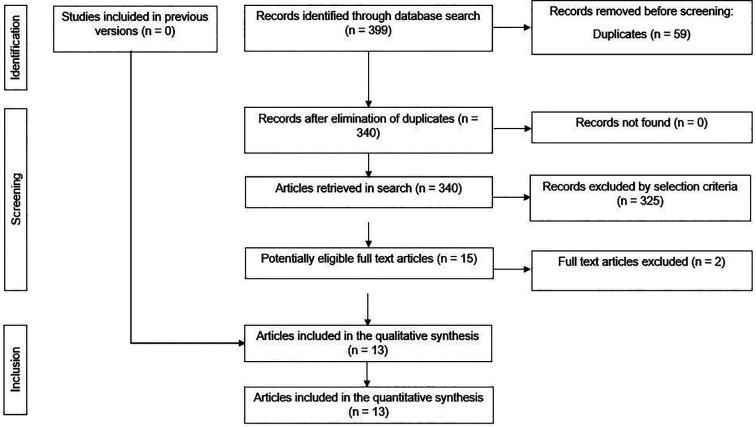
PRISMA diagram showing the process of inclusion and exclusion of studies in the systematic review.

The electronic and manual search strategy yielded a total of 399 articles, excluding 59 duplicates (Fig. 1). After screening by title and abstract, 15 potentially eligible full-text articles were selected. As a result, two studies were excluded (16,17), resulting in 13 randomised clinical trials (18-30) that met the eligibility criteria and were included for qualitative and quantitative synthesis. The reasons for study exclusion are listed in Table 2.

**Table 2:**
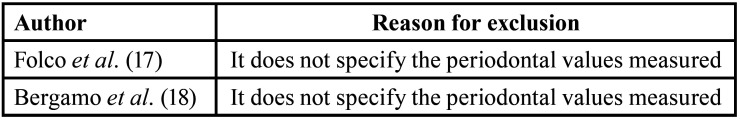
Reason for exclusion of studies.

- Characteristics of the studies included

Overall, 13 studies (19-31) from 7 different countries, all randomised clinical trials, were included. The number of patients included, age ranges, follow-up time and at least one of the values obtained for periodontal probing depth (PPD), plaque index (PI), gingival index (GI) and/or bleeding index (BI) were considered. It is important to note that, due to the different follow-up times, the final values from each of the studies were used (Table 3).

**Table 3:**
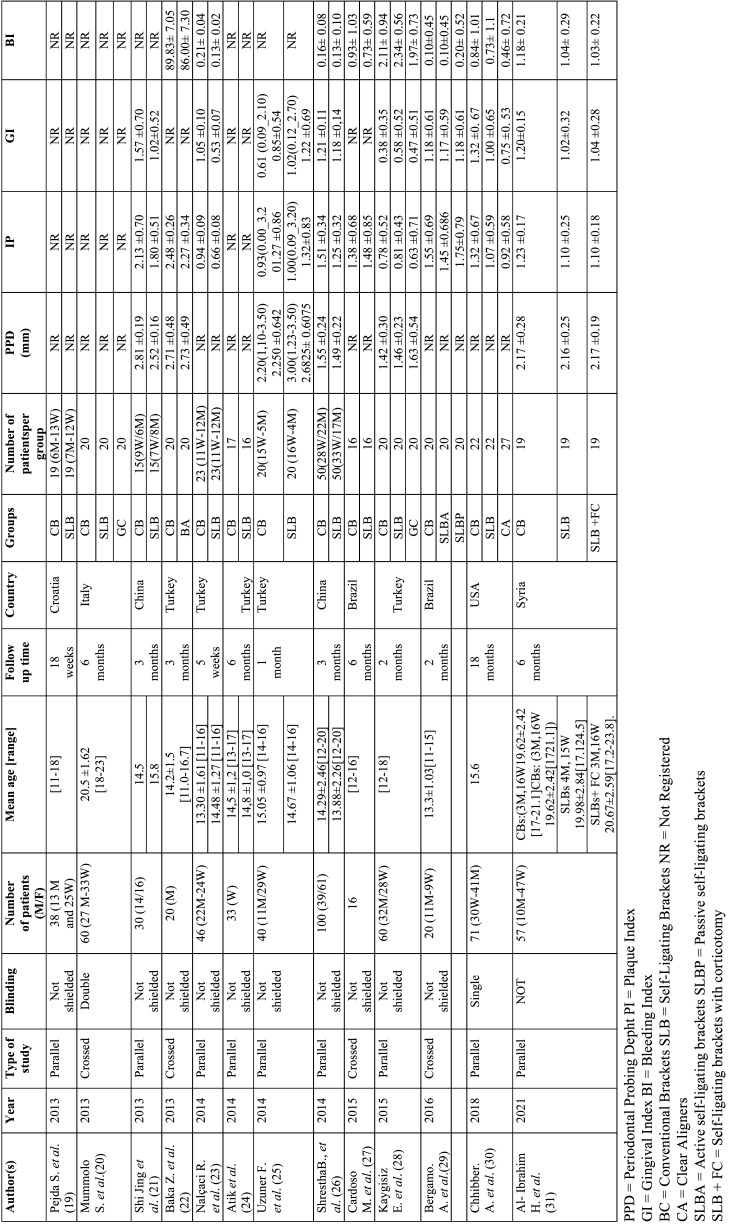
Characteristics of included studies.

- Risk of bias analysis of studies

**Figure 2:**
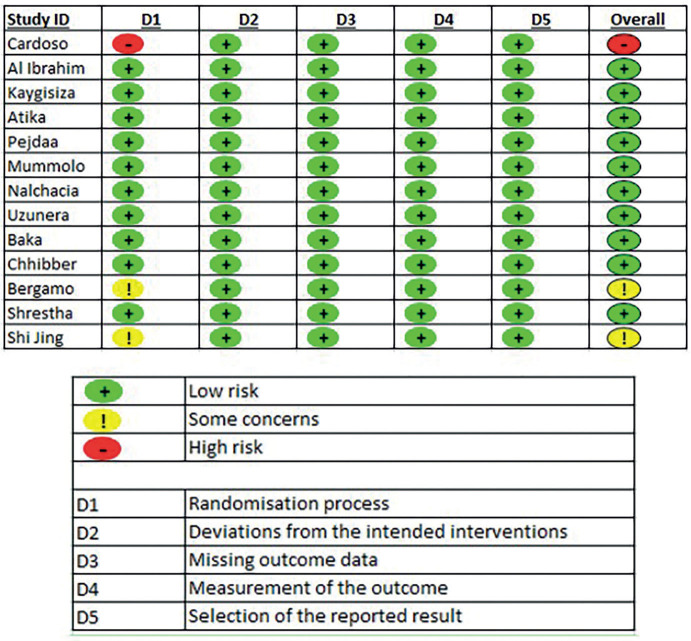
Risk of bias analysis of included studies.

In the data extraction process, 13 studies (19-31) were identified that met the inclusion criteria, and these studies were subjected to the risk of bias analysis for randomised clinical trials, which in general had a low risk of bias according to the 5 domains applied (Fig. 2).

- Synthesis of results (Meta-analysis)

**Figure 3:**
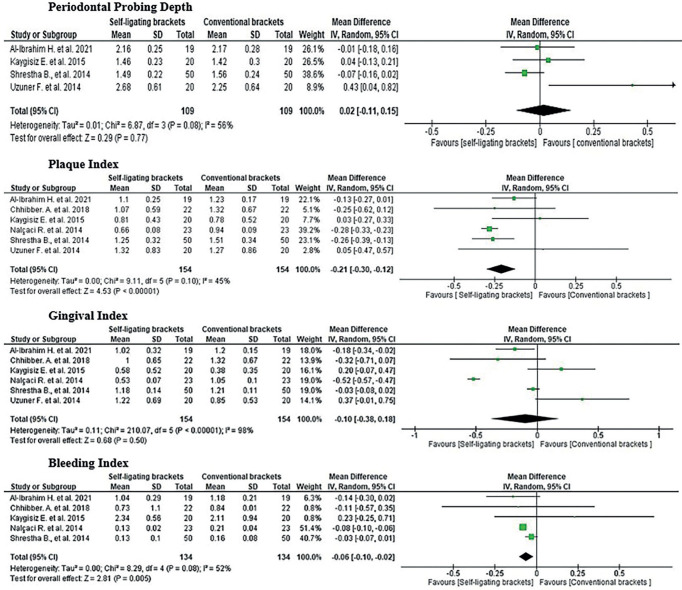
Meta-analysis.

The effect of conventional and self-ligating brackets on periodontal health was determined according to each of the evaluated indicators which are periodontal probing depth in 4 studies (25,26,28,31), plaque index in 6 studies (23,25,26,28,30,31), gingival index in 6 studies (23,25,26,28,30,31) and bleeding index in 5 studies (23,26,28,30,31), demonstrating advantages in the use of self-ligating brackets in plaque index and bleeding index, and no differences in comparison to self-ligating brackets with respect to PPD and GI. This is because plaque index indicated -0.21 with a confidence interval of -0.30, -0.12 and bleeding index -0.06 with a confidence interval of -0.10, -0.02 in favour of self-ligating brackets; while PPD showed a value of 0.02 with a confidence interval of -0.11, 0.15 and GI had -0.10 with a confidence interval of -0.38, 0.18. (Fig. 3).

Heterogeneity and final effect are also observed, where the I statistic2 is equal to 45% and 52% for plaque index and bleeding index respectively; which indicates that, if there is variability due to heterogeneity between studies, this is corroborated by the final effect which reflects a Z=4.53 with a *p*<0.00001 and Z=2.81 with a *p*=0.005 for the same indicators; which shows that there is a better periodontal condition with the use of self-ligating brackets with respect to plaque index and bleeding index .

- GRADE Analysis

When assessing the quality of evidence and grading the strength of recommendation for the included studies, it was observed that the analysis using the GRADE system yields a moderate level of certainty for the plaque index and bleeding index indicators, suggesting that the results of this study are likely to be in line with reality. However, the periodontal probing depth shows a low level of certainty, and the gingival index shows a very low level of certainty, indicating that the results may have discrepancies compared to reality (Table 4).

**Table 4:**
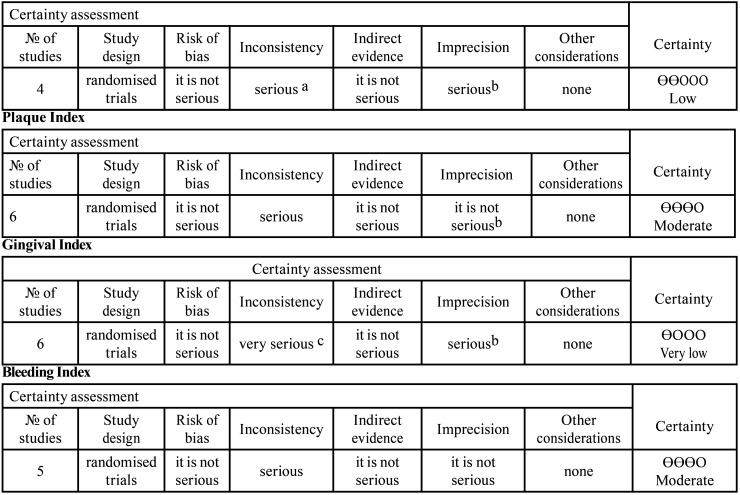
GRADE analysis.

## Discussion

The aim of the present systematic review and meta-analysis was to determine the effect on periodontal health of conventional brackets and self-ligating brackets. The findings suggest that self-ligating brackets may offer some advantages in terms of reducing the plaque index and bleeding rate, supported by a moderate level of certainty in the GRADE analysis. However, when assessing other indicators, such as periodontal probing depth and gingival index, no significant differences were observed between self-ligating and conventional brackets; furthermore, the GRADE analysis reflected low to very low certainty for these indicators. These results suggest that, while both types of brackets may contribute to periodontal disorders, self-ligating brackets may perform slightly better in certain aspects.

It is important to contextualise our results with respect to previous research. For example, a 2018 study by Arbildo *et al*. (32) found no significant differences in periodontal health between patients treated with conventional and self-ligating brackets. In contrast, our data suggest that there are advantages to the use of self-ligating brackets, particularly in relation to plaque index and bleeding rate. One possible explanation for this discrepancy could be that the elastics and ligature wires used in conventional brackets retain more plaque, which may result in increased bleeding compared to self-ligating brackets; although it must also be accepted that inevitably the use of brackets, conventional or self-ligating, will result in greater difficulty in hygiene that may subsequently lead to periodontal alterations.

On the other hand, Elkordy *et al*. (33) conducted a systematic review assessing the methodological quality of several studies using AMSTAR2 (A Measurement Tool to Assess Systematic Reviews 2), and concluded that there was insufficient evidence to demonstrate the superiority of self-ligating brackets on periodontal health. Our review also addressed the quality of the evidence. Of the 13 selected studies, 1 was excluded from the meta-analysis due to a high risk of bias (27), while 2 studies had some concerns in the risk of bias assessment (18,21), so these studies were not included in the meta-analysis. The study by Baka *et al*. (22), was not included in the meta-analysis because it was a crossover analysis.

Furthermore, it is relevant to consider that orthodontic movement itself does not seem to have a detrimental effect on the periodontium (34), however, our study emphasises the impact of brackets on plaque retention and inflammation during orthodontic treatment and suggests a slight advantage in the use of self-ligating brackets compared to conventional brackets.

There were difficulties in determining the periodontal effect caused by brackets due to the lack of standardisation of diagnostic criteria and variability in the presentation of clinical outcomes in the studies evaluated (17,18,22). Chhibber *et al*. (30) and Al-Ibrahim *et al*. (31) used different parameters to measure gingival bleeding, such as the papillary bleeding index (PBI), in contrast to the other authors who indicate the use of the bleeding index (23,26,28), which could influence the results.

The study by Chhibber *et al*. (30), in addition to studying conventional brackets and self-ligating brackets, evaluated clear aligners (CA), finding no differences in their effect on the periodontium. The effect on bone resorption is also similar in Almagrami’s study (35), where both fixed appliances and CA produce periodontal alterations at the alveolar bone level.

Additionally, it is important to note that Al-Ibrahim *et al*. (31) incorporated in their study an additional evaluation by comparing self-ligating brackets with and without corticotomy. The results indicated that the combination of self-ligating brackets with corticotomy could be effective in accelerating the correction of crowding, reducing orthodontic treatment time by 50 % for adult patients. However, Darwiche *et al*. (36), caution that the available evidence on the effectiveness of the effectiveness of corticotomy-assisted accelerated orthodontics is limited, although they suggest that this approach could accelerate treatment duration by 2.2 to 3 times compared to conventional orthodontics.

In addition to evaluating the risk of bias in the included studies using the Risk of Bias 2 (Rob 2.0) tool, graphs were generated to visualize the potential presence of publication bias. However, these graphs were not included in the final assessment because the number of studies per outcome did not meet the minimum threshold of 10, which limited the feasibility of conducting a robust statistical analysis. Although a formal analysis of publication bias could not be performed, its relevance is acknowledged, and it is suggested that future studies with a larger number of trials can address this important consideration in greater depth.

The heterogeneity observed among the studies included in this review could be attributed to variations in the follow-up periods. Despite this limitation, our findings point to certain benefits associated with the use of self- ligating brackets, which is corroborated by applying the GRADE system to the studies analysed. However, due to the variability in follow-up periods it is not possible to determine precisely when self-ligating brackets outperform conventional brackets in terms of improving periodontal conditions.

This systematic review and meta-analysis suggests that self-ligating brackets may offer some advantages in reducing plaque and bleeding rates compared to conventional brackets. However, it is essential to interpret these results with caution due to the heterogeneity between studies and the lack of standardisation in diagnostic criteria. Future research with more robust designs and standardised diagnostic criteria is needed to clarify the real impact of different types of brackets on periodontal health.

## Conclusions

Based on the results of the present systematic review and meta-analysis, it can be inferred that self-ligating brackets are likely to be more effective in preserving periodontal health compared to conventional brackets (plaque index and bleeding index). 

## References

[1] Papageorgiou SN, Höchli D, Eliades T (2017). Outcomes of comprehensive fixed appliance orthodontic treatment: A systematic review with meta-analysis and methodological overview. Korean J Orthod.

[2] Coronel-Zubiate FT, Marroquín-Soto C, Geraldo-Campos LA, Aguirre-Ipenza R, Urbano-Rosales LM, Luján- Valencia SA (2022). Association between orthodontic treatment and the occurrence of temporomandibular disorders: A systematic review and meta-analysis. J Clin Exp Dent.

[3] Singla S, Kamboj M, Gupta P, Lehl G, Talwar M (2022). Clinical evaluation of periodontal status in subjects with multibracket appliances and the role of age and gender during initial months of fixed orthodontic treatment. J Indian Soc Periodontol.

[4] Jiang Q, Li J, Mei L, Du J, Levrini L, Abbate GM (2018). Periodontal health during orthodontic treatment with clear aligners and fixed appliances. The Journal of the American Dental Association.

[5] Maizeray R, Wagner D, Lefebvre F, Lévy-Bénichou H, Bolender Y (2021). Is there any difference between conventional, passive and active self-ligating brackets? A systematic review and network meta- analysis. International orthodontics.

[6] Cattaneo PM, Tepedino M, Hansen EB, Gram AR, Cornelis MA (2022). Operating time for wire ligation with self-ligating and conventional brackets: A standardized in vitro study. Clinical and experimental dental research.

[7] Wagner D, Lévy-Benichou H, Lefebvre F, Bolender Y (2020). Are self-ligating brackets more efficient than conventional brackets? A meta-analysis of randomized controlled and split-mouth trials. Orthod Fr.

[8] Stöhr J, Barbaresko J, Neuenschwander M, Schlesinger S (2021). Bidirectional association between periodontal disease and diabetes mellitus: a systematic review and meta-analysis of cohort studies. Sci Rep.

[9] Papageorgiou SN, Hagner M, Nogueira AV, Franke A, Jäger A, Deschner J (2017). Inflammatory bowel disease and oral health: systematic review and a meta-analysis. J Clin Periodontol.

[10] O'Keeffe M, Kelly M, O'Herlihy E, O'Toole PW, Kearney PM, Timmons S (2019). Potentially modifiable determinants of malnutrition in older adults: A systematic review. Clin Nutr.

[11] Chen P, Hong F, Yu X (2022). Prevalence of periodontal disease in pregnancy: A systematic review and meta-analysis. J Dent.

[12] Humphrey LL, Fu R, Buckley DI, Freeman M, Helfand M (2008). Periodontal disease and coronary heart disease incidence: a systematic review and meta-analysis. J Gen Intern Med.

[13] Jepsen K, Sculean A, Jepsen S (2023). Complications and treatment errors involving periodontal tissues related to orthodontic therapy. Periodontol 2000.

[14] Ruf S, Proff P, Lisson J (2021). Zahn- und Kieferfehlstellungen - gesundheitliche Relevanz und Behandlung [Health relevance of malocclusions and their treatment]. Bundesgesundheitsblatt Gesundheitsforschung Gesundheitsschutz.

[15] Gehlot M, Sharma R, Tewari S, Kumar D, Gupta A (2022). Effect of orthodontic treatment on periodontal health of periodontally compromised patients. Angle Orthod.

[16] Papageorgiou SN, Antonoglou GN, Michelogiannakis D, Kakali L, Eliades T, Madianos P (2022). Effect of periodontal-orthodontic treatment of teeth with pathological tooth flaring, drifting, and elongation in patients with severe periodontitis: A systematic review with meta-analysis. J Clin Periodontol.

[17] Folco A, Benítez-Rogé S, Iglesias M, Calabrese D, Pelizardi C, Rosa A (2014). Gingival response in orthodontic patients: Comparative study between self-ligating and conventional brackets. Acta odontol latinoam.

[18] Bergamo AZN, Nelson-Filho P, Andrucioli MCD, do Nascimento C, Pedrazzi V, Matsumoto MAN (2017). Microbial complexes levels in conventional and self-ligating brackets. Clin Oral Invest.

[19] Pejda S, Varga ML, Milosevic SA, Mestrovic S, Slaj M, Repic D (2013). Clinical and microbiological parameters in patients with self-ligating and conventional brackets during early phase of orthodontic treatment. Angle Orthod.

[20] Mummolo S, Marchetti E, Giuca MR, Gallusi G, Tecco S, Gatto R (2013). In-office bacteria test for a microbial monitoring during the conventional and self-ligating orthodontic treatment. Head Face Med.

[21] Shi J, Liu Y, Hou J, Yan Z, Peng H, Chang X (2013). Comparison of periodontal indices and Porphyromonas gingivalis between conventional and self-ligating brackets. West China Journal of Stomatology.

[22] Baka ZM, Basciftci FA, Arslan U (2013). Effects of 2 bracket and ligation types on plaque retention: A quantitative microbiologic analysis with real-time polymerase chain reaction. Am J Orthod Dentofac Orthop.

[23] Nalçaci R, Özatb Y, Çokakoǧ Luc S, Tü Rkkahraman H, Önale S, Kaya S (2014). Effect of bracket type on halitosis, periodontal status, and microbial colonization. Angle Orthod.

[24] Atik E, Ciǧer S (2014). An assessment of conventional and self-ligating brackets in Class i maxillary constriction patients. Angle Orthod.

[25] Uzuner FD, Kaygisiz E, Çankaya ZT (2014). Effect of the bracket types on microbial colonization and periodontal status. Angle Orthod.

[26] Shrestha B, Jin X, Chen L, Shrestha R (2014). Comparative Study of Periodontal Status of Early Orthodontic Subjects treated with Self-ligating Brackets vs Conventional Edgewise Brackets. J Indian Orthod Soc.

[27] Cardoso M, Saraiva PP, Maltagliati LÁ, Rhoden FK, Costa CCA, Normando D (2015). Alterations in Plaque Accumulation and Gingival Inflammation Promoted by Treatment with Self-Ligating and Conventional Orthodontic Brackets. Dental Press J Orthod.

[28] Kaygisiz E, Uzuner FD, Yuksel S, Tanerd L, Çulhaoʇlu R, Sezgin Y (2015). Effects of self-ligating and conventional brackets on halitosis and periodontal conditions. Angle Orthod.

[29] Bergamo AZ, Nelson-Filho P, Romano FL, da Silva RA, Saraiva MC, da SilvaLA (2016). Gingival Crevicular Fluid Volume and Periodontal Parameters Alterations after Use of Conventional and Self-Ligating Brackets. Journal of Orthodontics.

[30] Chhibbe RA, Agarwal S, Yadav S, Kuo CL, Upadhyay M (2018). Which orthodontic appliance is best for oral hygiene? A randomized clinical trial. American Journal of Orthodontics and Dentofacial Orthopedics.

[31] Al-Ibrahim HM, Hajeer MY, Alkhouri I, Zinah E (2022). Leveling and alignment time and the periodontal status in patients with severe upper crowding treated by corticotomy-assisted self-ligating brackets in comparison with conventional or self-ligating brackets only: a 3-arm randomized controlled clinical trial. J World Fed Orthod.

[32] Arbildo H, Gamarra L, Rojas S, Infantes E, Vàsquez CF (2018). Comparing the periodontal clinical effect between conventional and self-ligating brackets: systematic review and meta-analysis. J Oral Res.

[33] Elkordy S, Palomo L, Palomo J, Mostafa Y (2019). Do fixed orthodontic appliances adversely affect the periodontium? A systematic review of systematic reviews. Seminars in Orthodontics.

[34] Martin C, Celis B, Ambrosio N, Bollain J, Antonoglou GN, Figuero E (2022). Effect of orthodontic therapy in periodontitis and non-periodontitis patients: a systematic review with meta-analysis. J Clin Periodontol.

[35] Almagrami I, Almashraqi AA, Almaqrami BS, Mohamed AS, Wafaie K, Al-Balaa M (2023). A quantitative three-dimensional comparative study of alveolar bone changes and apical root resorption between clear aligners and fixed orthodontic appliances. Prog Orthod.

[36] Darwiche F, Khodari E, Aljehani D, Gujar AN, Baeshen HA (2020). Comparison of effectiveness of corticotomy- assisted accelerated orthodontic treatment and conventional orthodontic treatment: a systematic review. J Conte Dent Pr.

